# Development of Pork Ball Freshness Monitoring Labels Based on Carrageenan/Polyvinyl Alcohol Matrices and Anthocyanins from Three Petals: A Comparative Study

**DOI:** 10.3390/foods15162769

**Published:** 2026-08-07

**Authors:** Wenzheng Zhu, Xinyu Zhang, Beining Zhang, Yan Xu, Huimin Yong, Jun Liu, Xiaoyan Zhou

**Affiliations:** 1 College of Tourism and Culinary, Yangzhou University, Yangzhou 225127, China; zhuwz@yzu.edu.cn (W.Z.); 13962861789@163.com (X.Z.); lzy1853699631@163.com (B.Z.); 2 Engineering Research Center for Huaiyang Cuisine of Jiangsu Province, Yangzhou 225127, China; 3 Key Laboratory of Chinese Cuisine Intangible Cultural Heritage Technology Inheritance, Ministry of Culture and Tourism, Yangzhou University, Yangzhou 225127, China; 4 College of Food Science and Engineering, Yangzhou University, Yangzhou 225127, China; xuyanlp@yzu.edu.cn (Y.X.); 008694@yzu.edu.cn (H.Y.); junliu@yzu.edu.cn (J.L.); 5 College of Culinary Arts and Hotel Management, Shanghai Zhongqiao Vocational and Technical University, Shanghai 201514, China

**Keywords:** anthocyanins, flower source, intelligent film, pork ball, freshness monitoring

## Abstract

Anthocyanins were extracted from the petals of *Loropetalum chinense* var. *rubrum*, *Petunia hybrida*, and *Rhododendron pulchrum* Sweet, denoted as LA, PA and RA, respectively. Physicochemical properties of *Loropetalum chinense* var. *rubrum* (LA), *Petunia hybrida* (PA) and *Rhododendron pulchrum* Sweet (RA) were comparatively evaluated. Among them, RA exhibited the highest total anthocyanin content (49.41 ± 0.23^a^ mg/g) along with the total phenolic amount (102.01 ± 0.15^a^ mg/g). Immobilizing the obtained anthocyanin-rich extract in a carrageenan (CP) and polyvinyl alcohol (PVA) matrix and drying it at 30 °C and 50% relative humidity yielded a film intended for use as a smart packaging label to monitor Pork ball freshness. Scanning electron microscopy (SEM), X-ray diffraction (XRD), and fourier transform infrared spectroscopy (FTIR) analyses demonstrated excellent compatibility between anthocyanins and CP/PVA matrices, resulting in dense structures. The results of physicochemical and functional properties revealed that the prepared control films and anthocyanin films containing RA (CP-RA film) showed optimal performance, including superior visible light/oxygen barrier properties, tensile strength (45.49 MPa), elongation at break (26.47%), antioxidant capacity (42.75%), and pH-sensitivity. In addition, CP-RA film maintained its superior color stability over various temperature situations and displayed high sensitivity to changes in total volatile basic nitrogen (TVB-N) values.

## 1. Introduction

Traditional petroleum-based polymers, including polyethylene, polypropylene, and polyethylene terephthalate, constitute the dominant 70% of raw materials used in packaging production. Plastic pollution has increased significantly as a result of plastic’s inability to decompose. Due to its ability to effectively block moisture, oxygen, and mechanical stress, food packaging has been recognized as indispensable. In recent years, the packaging industry has increasingly focused on sustainability and environmental awareness, which has propelled the innovation of biodegradable plastics. Therefore, there is an urgent need for modern packaging technologies to meet these demands. In the rapidly evolving field of food packaging, developing green and biodegradable functional materials to increase food’s shelf life has attracted attention [1].

While preserving the contents’ quality and safety, active packaging engages with the package’s internal environment. Improving food’s shelf life is the purpose of these studies, preserving its freshness, appearance, and nutritional content [2]. However, the ability to promptly monitor the freshness of food is limited by these bioactive packaging materials. Meat spoilage not only leads to a noticeable change in taste but also generates a large number of viruses and bacteria, which are harmful to human health and well-being [3]. Therefore, it is urgent to develop intelligent packaging films with real-time monitoring capabilities, so as to visually reflect the freshness changes in Pork balls. This device is used to keep an eye on low-temperature-kept and refrigerated foods [4]. Under normal circumstances, smart food packaging films with pH indicators comprise two parts: the film matrix and the pH indicator. Nowadays, the capacity for rapid monitoring is the key factor determining the application value of intelligent packaging systems.

Using anthocyanins from different sources, researchers have developed various intelligent color-changing films. Factors that influence the performance of these films include the origin, dosage, and purity of anthocyanins [5]. Carrageenan (CP), a primary cell wall component of red algae, is a well-established gelling agent and thickener. Among polysaccharides, it is considered one of the most effective high-molecular-weight materials meeting food packaging standards [5,6]. It possesses renewability, biodegradability, good biocompatibility, and film-forming properties. Polyvinyl alcohol (PVA) films exhibit good gas barrier properties, preventing the permeation of oxygen and other gases. *Loropetalum chinense* var. *rubrum* is native to tropical and subtropical regions. Its leaves have vivid colors and possess strong adaptability to the environment. It is also resistant to pruning and easy to shape. The plant contains various bioactive components such as anthocyanins and has certain medicinal value. *Petunia hybrida* belongs to the Solanaceae family and is a perennial herb. Due to its rich color variety, unique flower shape, and long flowering period, it has become a highly valuable ornamental variety in landscape greening. The diverse colors and long-lasting flowering period make it widely used in global landscape design. The anthocyanin content of *Rhododendron pulchrum* Sweet is high, the resources are abundant, and the color is bright. It is a promising new resource for developing natural anthocyanin-based pigments and has broad application prospects. Currently, the research on anthocyanin smart membranes mainly focuses on common fruits and vegetables, such as purple sweet potatoes, purple cabbage, and blueberries. However, there have been few systematic studies on flower-derived anthocyanins. This research systematically compares three representative flower sources (LA, PA, and RA) under the same conditions, filling the research gap in the field of smart packaging for flower-derived anthocyanins. Moreover, it provides experimental evidence to support the use of flowers as a long-neglected but highly potential source of anthocyanins. Conducting such studies can not only elevate the profile of plant resources such as LA, PA, and RA, but also help identify superior anthocyanin-producing species. This will facilitate their rational development and conservation, promoting the sustainable utilization of plant resources. The three have distinct color manifestations, possibly containing various types of anthocyanin components. Selecting them as research subjects can increase the probability of obtaining different structures and properties of anthocyanins, thereby providing a more abundant sample support for the comparative study of anthocyanins. Most existing studies merely regard anthocyanins as film colorants, with few confirming them as the key factor regulating the comprehensive performance of intelligent films, and rarely seeing their application in the shelf-life monitoring of Chinese-style pork balls. To fill these gaps, this study compared the differences in film formation by various anthocyanins, characterized the structure, physicochemical and functional properties of anthocyanins and the composite films, and preliminarily explored the feasibility of the as-prepared indicator film for real-time freshness monitoring of cooked meat products.

## 2. Materials and Methods

### 2.1. Materials, Chemicals and Reagents

*Loropetalum chinense* var. *rubrum* and *Rhododendron pulchrum* Sweet were collected from the campus of Yangzhou University; *Petunia hybrida* was sourced from Yangzhou. The flowering periods of the three plants are concentrated in the middle to late April. Fresh flower materials can be collected simultaneously to ensure consistency of the materials. Ethanol, hydrochloric acid (HCl), Folin–Ciocalteu reagent, sodium carbonate, sodium acetate, potassium chloride, disodium hydrogen phosphate, citric acid, glycine, sodium hydroxide, PVA (degree of polymerization: ~1750), CP, glycerol, 2,2-diphenyl-1-picrylhydrazyl (DPPH), ammonia water, and boric acid were bought from Shanghai Macklin Biochemical Co., Ltd., Shanghai, China.

### 2.2. Extraction and Characterization of Anthocyanins

#### 2.2.1. Anthocyanin Extraction

Different flower petals were separately freeze-dried, pulverized, subsequently immersed in an aqueous solution of 80% ethanol with 0.5% HCl [7], and extracted overnight at 4 °C after juicing. After the ethanol was extracted from the mixture by rotary evaporation at 45 °C, the concentrate was spun up for 15 min at 12,000 rpm. After re-evaporation and centrifugation, the supernatant was vacuum-dried at 50 °C for 72 h to obtain anthocyanin extract powder. The anthocyanin extracts from *Loropetalum chinense* var. *rubrum*, *Petunia hybrida* and *Rhododendron pulchrum* Sweet were named as LA, PA, and RA, respectively.

#### 2.2.2. Determination of Anthocyanin Content

Different amounts of sample (2–5 mg) were weighed and dissolved in equal volumes of pH 1.0 and pH 4.5 buffers to prepare 1 mg/mL solutions. Each solution was then diluted 10-fold and 50-fold with buffer, respectively, and allowed to equilibrate for two hours in the dark. The wavelength of maximum absorption was used to measure the absorbance value at 700 nm of the sample [8]. The extraction amount of anthocyanins was calculated by the pH differential method:

(1) $$Anthocyanin\;content\;(mg/g)\;=\;\frac{A\times \mathrm{Mw}\times V\times \mathrm{DF}}{\varepsilon \mathrm{LW}}$$

where A is (A_λmax_ − A_700nm_) × pH1.0 − (A_λmax_ − A_700nm_) × pH4.5, Mw is the molar mass of cyanidin-3-O-glucoside (449.2 g/mol), DF is the dilution factor, V is the final volume of the sample to be tested (mL), ε is the extinction coefficient of cyanidin-3-O-glucoside (26,900 L·mol^−1^ cm^−1^), L is the optical path length of the cuvette (1 cm), and W is the sampling weight (g).

#### 2.2.3. Determination of Total Phenol Content

Anthocyanin powder (2–5 mg) was dissolved in distilled water to prepare a sample solution with a concentration of 1 mg/L. The Folin phenol reagent was diluted 10-fold in advance. Subsequently, 1 mL of sample solution and 1 mL of Folin diluent were taken and mixed evenly; the mixture was allowed to react in the dark at 30 °C for 5 min. Then 5 mL of saturated Na_2_CO_3_ solution was added and mixed evenly. After standing for 2 h, the absorbance was measured at 760 nm. A standard curve of gallic acid was prepared at concentrations of 0.01, 0.02, 0.03, 0.04, and 0.05 mg/mL and used to determine the total phenolic content of the samples. The regression equation of the obtained standard curve is as follows: y = 7.5914x + 0.0399 (R^2^ = 0.9853).

#### 2.2.4. pH Sensitivity

To create a 1 mg/mL solution, the 2–5 mg extract was placed in distilled water and mixed with different pH buffers (3–12) at a 1:1 ratio; each anthocyanin’s color sensitivity in different pH conditions was photographed.

### 2.3. Preparation of CP-PVA-Anthocyanin Blend Films

Based on the method of Liu et al. and Yong et al. [9,10], different CP-PVA-based films containing anthocyanin were prepared as follows. CP (3.4 g) was dissolved in distilled water at 95 °C with stirring for 1 h to obtain a CP solution. Similarly, 3.4 g of PVA was dissolved at 95 °C with stirring for 1 h to obtain a PVA solution. The CP solution (210 mL) was mixed with 68 mL of the PVA solution at 95 °C for 2 h, yielding a total volume of 278 mL and a final polymer concentration of approximately 2.45% (*w*/*v*). The resulting CP-PVA solution was then combined with 1 wt% anthocyanins and 30 wt% glycerol (based on the CP-PVA matrix), corresponding to approximately 0.245 mg/mL of anthocyanins in the solution. This 1 wt% anthocyanin dosage was determined as the optimal concentration via our preliminary optimization experiments and the reference of Liu et al. [9]. Films loaded with 1 wt% anthocyanins possessed balanced antioxidant capacity and obvious pH-triggered color shifts, which satisfied the requirements of active and intelligent food packaging. Subsequently, the film-forming solution was degassed, and approximately 278 mL of it was poured into a self-designed acrylic plate (24 cm × 24 cm) and dried into a film at 30 °C and 50% relative humidity until constant weight was achieved. The prepared blank film and the anthocyanin films containing LA, PA, and RA were designated CP film, CP-LA film, CP-PA film, and CP-RA film, respectively. Before testing, all films were conditioned in a desiccator at 25 °C and 50% relative humidity for 2 days.

### 2.4. Characterization of Thin Films

#### 2.4.1. SEM

Film samples were fractured in liquid nitrogen and then sputter-coated with gold under vacuum. The cross-sectional morphology of anthocyanin-incorporated films was observed using a scanning electron microscope (Gem­-iniSEM 300, Carl Zeiss, Jena, Germany) at an accelerating voltage of 5 kV.

#### 2.4.2. FT-IR Spectra

The infrared absorption spectra of the films were determined using an FT-IR Spectrometer equipped (Cary 670, Varian, Palo Alto, CA, USA) with an Attenuated Total Reflectance (ATR) accessory, throughout a 4000–400 cm^−1^ range. Each sample was scanned three times, and the data were collected for analysis.

#### 2.4.3. XRD Analysis

A polycrystalline X-ray diffractometer was used to ascertain the crystallographic properties of anthocyanin mixed films. Test conditions: voltage 40 kV, current 40 mA and 2°/min scanning between 5° and 65° to derive the XRD pattern.

### 2.5. Determining Thin Films Chemical and Physical Indices

#### 2.5.1. Color and Light Transmittance

Color parameters (L, a, b values) of each film sample were measured in triplicate using an SC-80C colorimeter (Kang guang, Beijing, China). The total color difference (ΔE) was calculated according to the corresponding formula. Meanwhile, photographs of the films were captured with a camera [11].

(2) $$\Delta E\;=\;\sqrt{(L^{*}-L{)}^{2}\;+\;(a^{*}-a{)}^{2}\;+\;(b^{*}-b{)}^{2}}$$

where L*, a*, and b* are the color values of the standard white plate (L* = 93.28, a* = 1.1, b* = 1.3).

Using a Lambda 35 spectrometer (Perkin-Elmer, Waltham, MA, USA), scan the film samples across the 200–800 nm wavelength range to find their light transmittance.

#### 2.5.2. Thickness

A random selection of ten points was made for each film. The thickness was measured using a micrometer (Bonthe, Shangqiu, China) with a 0.01 mm accuracy, and the results were recorded as d_1_, d_2_, d_3_, …, d_10_. The following formula was used to determine the films’ thickness (taking the average of ten values).

(3) $$Thickness\;=\;\frac{d_{1}\;+\;d_{2}\;+\;d_{3}\;+\;\ldots \ldots \;+\;d_{10}}{10}$$

#### 2.5.3. Water Vapor Permeability (WVP)

Following the procedure of Yong et al. [10], the film samples (6 cm × 6 cm) were sealed in a centrifuge tube with the mouth filled with dry silica gel (100% RH) and put in a desiccator. The silica gel was weighed every 24 h at 20 °C. The measurement was continuously carried out for 7 days. Triplicate measurements determined the WVP of each film. The WVP of the film was calculated according to the following formula, and the result was expressed as gm^−1^ s^−1^ Pa^−1^:

(4) $$WVP\;=\;\frac{W\;\times \;x}{t\;\times \;A\;\times \;∆P}$$

where W is the mass of water penetrating through the film (g), x is the film thickness (m), A is the effective permeation area of water vapor (m^2^), t is the time for water to penetrate, and ΔP represents the water vapor pressure differential on both ends of the film (2339 Pa).

#### 2.5.4. Water Contact Angle (WCA)

For WCA measurement, 2 μL of distilled water was carefully placed onto each indicator. High-definition microscope images of water droplets were captured using a video microscope (Gaopin, Suzhou, China) equipped with a charge-coupled device camera (GP-50, Gaopin Corp., Suzhou, China). The microscope was mounted horizontally and illuminated by an LED cold light source, with the entire process completed at 5 s intervals. After the image was taken, the WCA value was examined using ImageJ software (version 1.52, National Institutes of Health, Bethesda, MA, USA). Each indicator underwent this analysis procedure six times [12,13].

#### 2.5.5. Oxygen Permeability (OP)

The thickness of each sample was measured, and then it was fixed on the Basic 201 gas permeation tester (Labthink Corp., Jinan, Shandong, China). A 10 cm membrane disk was positioned between the gas permeation chambers. Using a vacuum pump (SHZ, Shanghai, China), all oxygen was totally removed from the gas permeation tester prior to testing. All tests were carried out at 23 °C and 50% RH [14]. The OP values for each film were obtained through three measurements.

#### 2.5.6. Mechanical Property

The determination of tensile strength and elongation at break referred to the method of Liu et al. [15], where a pre-cut rectangular film (60 mm × 10 mm) was fixed between two metal grips of a texture analyzer, with the initial grip distance set to 60 mm and the stretching rate set to 60 mm/min. Each test was repeated at least five times. The tensile strength and elongation at break were calculated via the formula below:

(5) $$TS\;=\;\frac{F}{W\;\times \;x}$$

where F is the tensile strength (N), W is the width of the film (mm), and x is the thickness of the film (mm).

(6) $$EAB\;(\%)\;=\;\frac{L}{L_{0}}$$

where L is the elongation of the film at break (mm), and L_0_ is the original length of the film (mm).

#### 2.5.7. Thermogravimetric Analysis (TGA)

TGA was conducted according to the method described by Yun et al. [14]. The test was conducted from 50 °C to 700 °C. The membrane sample (2 mg) was assessed in a nitrogen environment at a rate of 20 mL/min.

### 2.6. Functional Properties of the Films

#### 2.6.1. Antioxidant Activity

The DPPH radical scavenging assay was used to assess the film’s antioxidant activity [16]. A 10 mg film sample was mixed with 4 mL of 100 μmol/L ethanolic DPPH solution, and the mixture was incubated at 20 °C for 1 h. Afterward, the absorbance of the reaction solution was determined at 517 nm.

(7) $$\mathrm{Antioxidant}\;\mathrm{activity}\;(\%)\;=\;\frac{A_{0}-A_{1}}{A_{0}}\;\times \;100$$

where *A*_0_ is the absorbance of the blank solution, and *A*_1_ is the absorbance of the reaction solution.

#### 2.6.2. pH Sensitivity and Ammonia Sensitivity

The colorimetric response of the films in pH 3–12 buffer solutions was determined with modifications to the method reported by Wang et al. [17]. The film samples were immersed in the pH buffer solutions for a duration of 1 min. Subsequently, the film samples were taken out, and after the colors were noted, their surfaces were dried with absorbent paper. To evaluate the films’ ammonia sensitivity, for six hours, the color changes were observed after they were additionally subjected to a 1 mol/L ammonia solution.

#### 2.6.3. Stability

Stability analysis was carried out under environmental conditions of 4 °C and 20 °C, and the test period was 30 days. Every three days, we measured each index’s color and Δ*E* value fluctuations. Each index was subjected to three analyses [13].

### 2.7. CP-PVA-Anthocyanin Film as a Spoilage Indicator

Marbled pork consisting of lean muscle and adipose tissue at a mass ratio of 1:1 was minced. Scallions, ginger, rice wine and salt were added, followed by thorough stirring. Water starch was then incorporated and mixed uniformly. The obtained mixture was formed into dumplings of proper size. The dumplings were put into boiling water for shaping and subsequently simmered on low heat for 2 h.

The monitoring of the spoilage process of Pork balls referred to the method of Yun et al. [14] and was slightly adjusted; 150 g of Pork balls were placed in a 500 mL fresh-keeping box, and the anthocyanin-blended films (2 cm × 2 cm) were attached to the top of the space. The total volatile basic nitrogen value (TVB-N) and the film’s color change were recorded every two days during the 16 days of continuous monitoring at 4 °C. Three repetitions were performed at each time point.

### 2.8. Statistical Analysis

A total of 4 film groups (CP film, CP-LA film, CP-PA film, and CP-RA film) were set up in this study. Three parallel film samples were prepared for each group. For the freshness detection experiment of Pork balls, 3 specimens were used from each group and continuously monitored at 4 °C for 16 days. All data sets were pre-tested for normality and homogeneity of variance. When the data were normal or approximately normal, the results were expressed as mean ± standard deviation. One-way analysis of variance (ANOVA) followed by Duncan’s multiple range test was performed using SPSS 13.0 software. Significance was considered when *p* < 0.05.

## 3. Results and Discussion

### 3.1. Characterization of Anthocyanins

**Figure 1:**
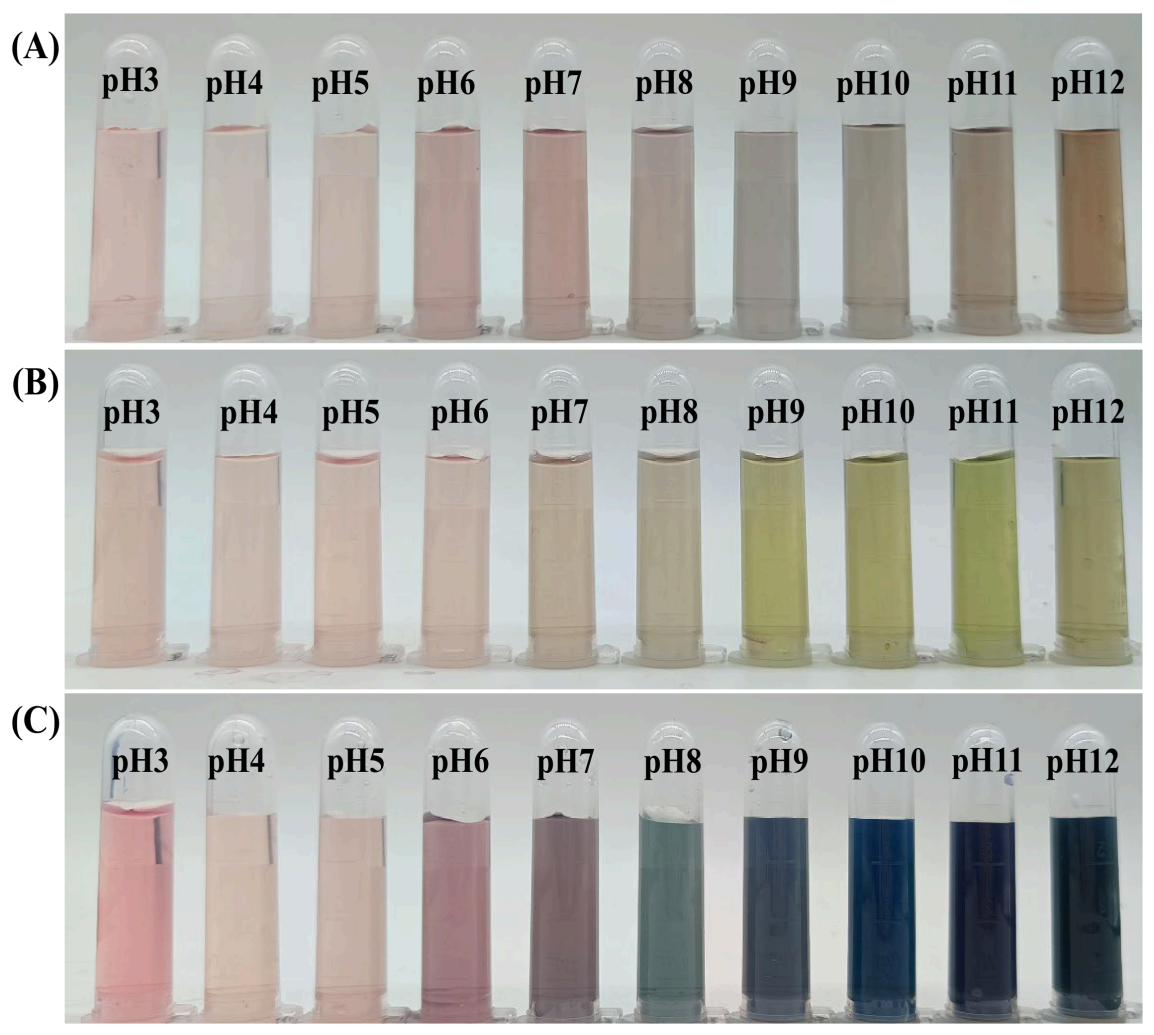
The color changes in LA (**A**), PA (**B**) and RA (**C**) in different pH buffer solutions.

**Figure 2:**
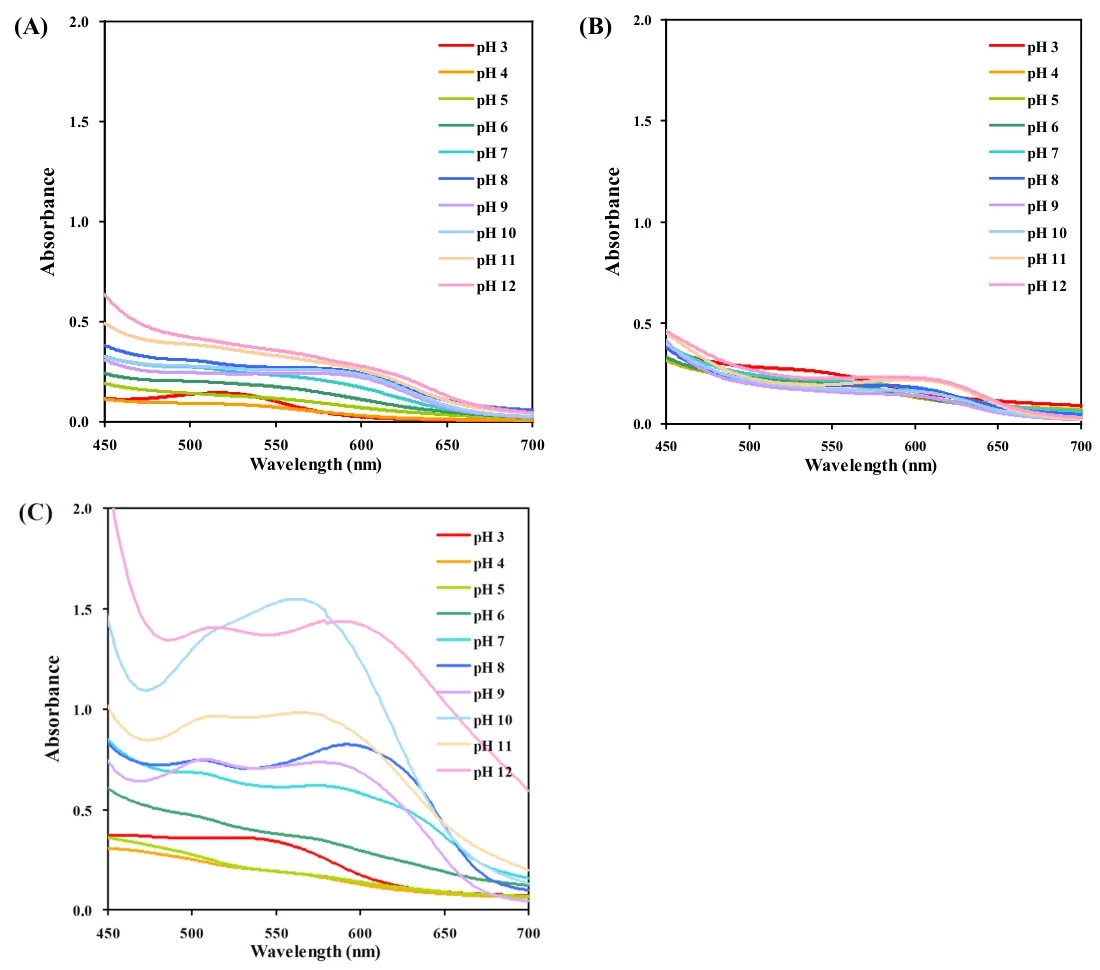
The ultraviolet-visible spectra of LA (**A**), PA (**B**) and RA (**C**).

The total anthocyanin content and total phenol content in LA, PA and RA were quantitatively analyzed by the pH differential method. At 49.41 ± 0.23^a^ mg/g and 102.01 ± 0.15^a^ mg/g, respectively, RA had the greatest levels of total anthocyanin and total phenol. Next was LA, with 18.35 ± 0.17^b^ mg/g and 98.90 ± 0.07^b^ mg/g, respectively. The content of PA was the lowest, which was 16.30 ± 0.36^c^ mg/g and 68.64 ± 0.22^c^ mg/g, respectively. Differences existed in the total anthocyanin content and total phenol content of different types of petals. Figure 1 displayed how the colors of LA, PA, and RA changed in various pH buffer solutions. Upon increasing the pH of the buffer solution, the LA anthocyanin solution’s pink color eventually gave way to purple, gray, and brown. The color change in PA transitioned from pink to gray, green and yellow-green. Additionally, the RA anthocyanin solution’s hue varied from pink to light pink, purple, blue, and green in turn. The kinds and concentrations of anthocyanins had a direct correlation with these color changes. The color changes in anthocyanin solutions observed were slightly different from those reported in a large body of literature [14,18], which might be attributed to the differences in petal growth conditions and types. The main reason for anthocyanins’ intense coloring was their chemical structure. Its skeleton’s eight conjugated double bonds created a chromophore that could absorb light waves between 500 and 600 nm in wavelength, giving anthocyanins their various colors, including red, blue, purple, and gray. Anthocyanins were comparatively stable in acidic conditions, mostly existing as flavylium cations and exhibiting a red appearance [19]. When the pH value of the solution increased to 4–6, flavylium cations were transformed into gray or colorless methanol pseudobase through deprotonation. Under slightly neutral conditions (pH value of 6–8), anthocyanins’ structure changed from methanol pseudobase to quinoidal base, and their hue changed from pink to purple or blue. As the pH value further increased to alkaline (pH > 8), anthocyanins formed a yellow chalcone structure. The color intensity of anthocyanins was not only affected by their own structure, but also by external factors such as pH value, temperature, ultraviolet radiation and oxygen [20,21]. Figure 2 presents the changes in ultraviolet-visible absorption spectra of LA, PA and RA in different buffer solutions. The ultraviolet-visible absorption spectra confirmed the structural changes and color transitions of anthocyanins under different pH conditions. Among them, the structural change in the ultraviolet-visible absorption spectra of RA was the most significant, which might be due to the fact that anthocyanins of RA had a sensitive acid-base response mechanism.

### 3.2. Structural Characteristics of the Films

#### 3.2.1. SEM

**Figure 3:**
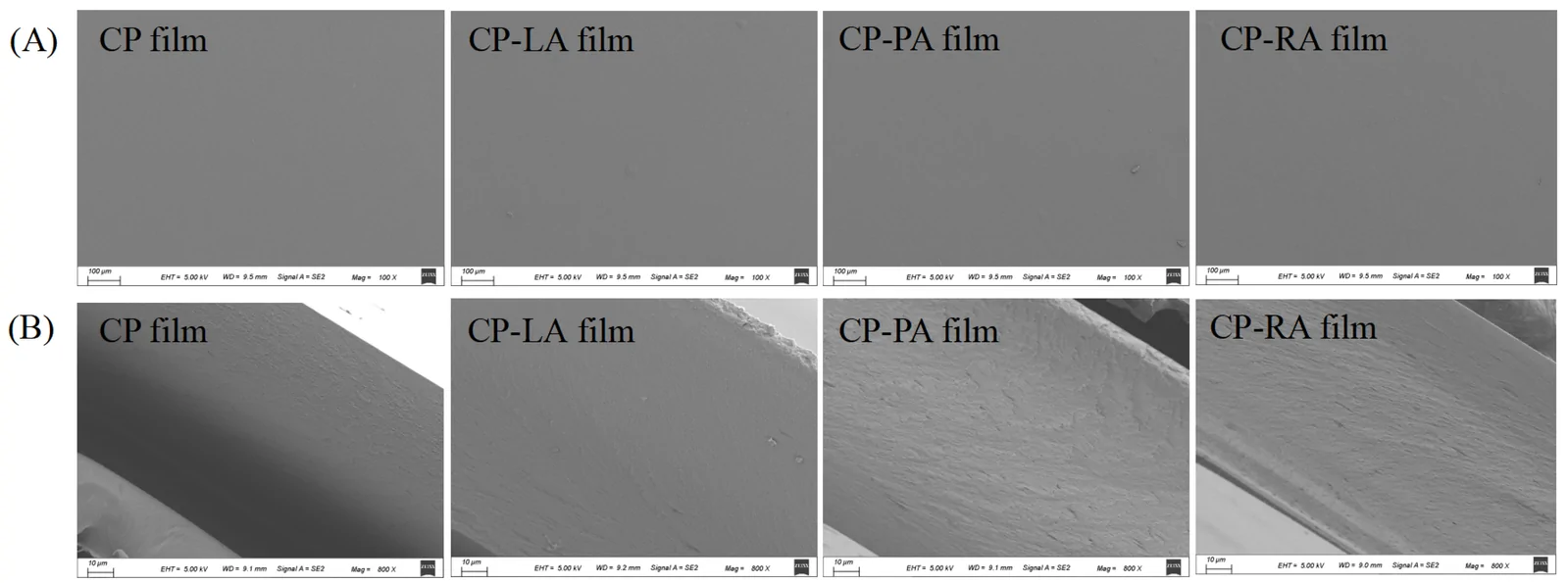
The surface and cross-sectional morphologies of (**A**) CP film, CP-PA film, CP-LA film, CP-RA film; (**B**) CP film, CP-PA film, CP-LA film, CP-RA film.

SEM testing was used to investigate the possible effects of anthocyanins on the films’ microstructure. According to the SEM pictures, all of the films’ surfaces had consistent, smooth features (Figure 3A,B). In Figure 3A, the surface of the CP film is the smoothest and flattest, with no obvious particles or protrusions. The CP-LA film surface has only a very small number of tiny, scattered protrusions, and the overall uniformity remains high. The CP-PA film and CP-RA film surfaces exhibit a few sparsely distributed tiny particle protrusions, but no agglomeration or defects are formed. In Figure 3B, the cross-section of the CP film shows a highly dense, uniform, homogeneous continuous structure with no layered structure or pores. The cross-section of the CP-LA film still maintains a dense and continuous structure, with only a very small number of tiny dispersed particles. The CP-PA film cross-section shows obvious layered ripple-like textures, with slightly reduced structural density. The CP-RA film cross-section presents a rough, irregular texture, with density between that of CP-LA and CP-PA. This observation indicated that LA, PA, and RA had good compatibility with the CP/PVA film matrix [22]. By comparing the thicknesses of the blank film and the anthocyanin-containing films, it was discovered that the film thickness increased when anthocyanins were added. Specifically, in ascending order of the increase in film thickness, they were the CP-LA film, CP-PA film and CP-RA film. This finding provided new insights into the application of anthocyanins in film materials and emphasizes their important role in regulating the physical properties of the films.

#### 3.2.2. FT-IR Spectra

**Figure 4:**
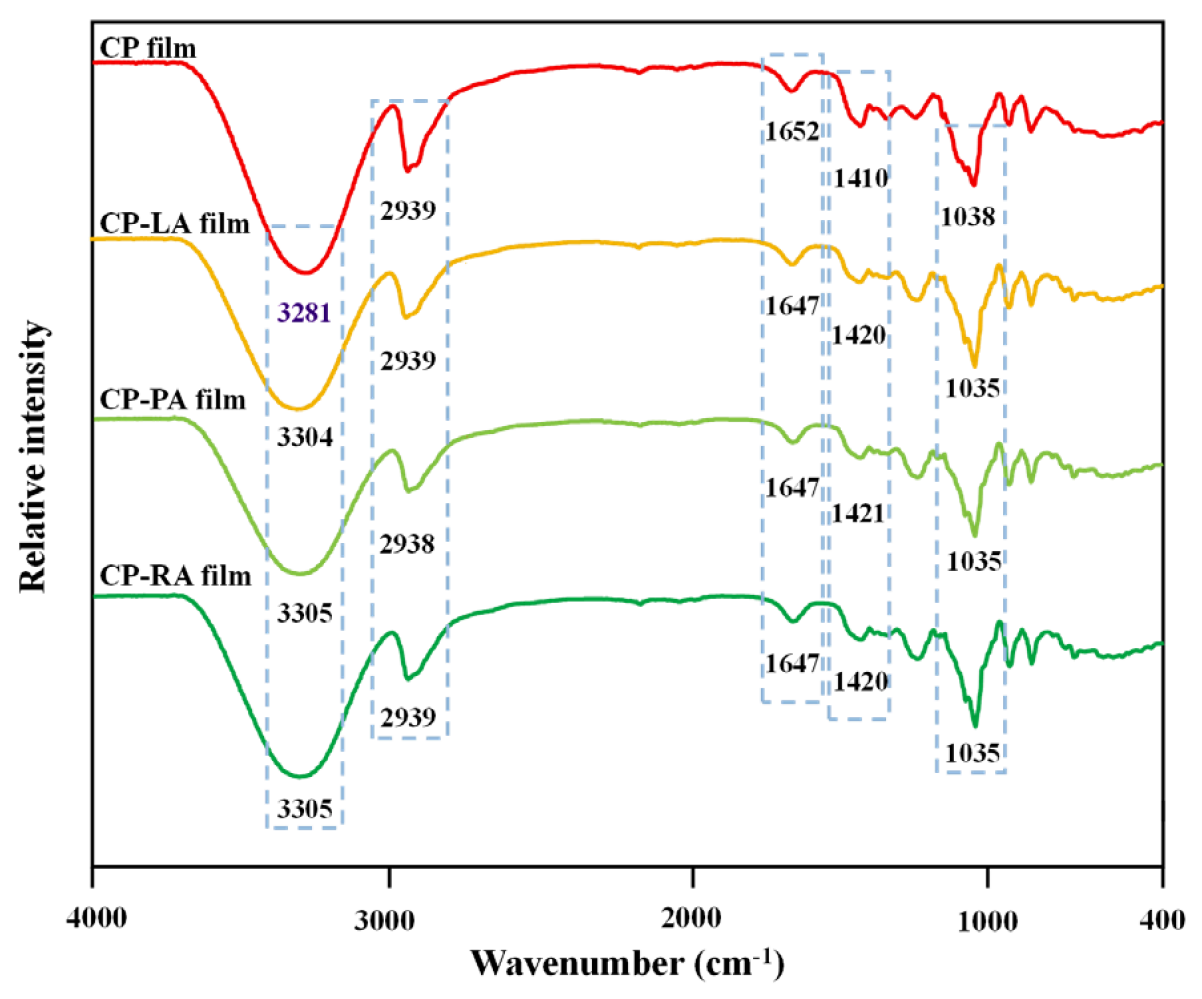
The FT-IR spectra of CP film, CP-PA film, CP-LA film, CP-RA film.

The functional groups suggesting the film components and their interactions were further disclosed by the FT-IR spectra. Figure 4 shows that the three anthocyanin-containing films and the blank film had some common characteristic bands. For example, there were O-H stretching vibrations at 3281–3305 cm^−1^, C-H stretching vibrations at 2938–2939 cm^−1^, combined water peaks at 1647–1652 cm^−1^, CH-CH_2_ bending vibrations at 1410–1421 cm^−1^, and C-O-C vibrations at 1035–1038 cm^−1^ [23,24]. Following the inclusion of anthocyanins, the O-H stretching vibrations and combined water peaks of the CP/PVA films shifted to higher wavelengths. The presence of polyphenols seems to have changed the molecular connection mode between CP and PVA. It is preliminarily speculated that this might be related to the physical cross-linking effect formed between the polyphenols and the matrix. It should be noted that this inference is based solely on the changes in the FTIR spectrum and lacks direct molecular-level evidence to support it [14]. Overall, the absorption peaks of the characteristic bands of the three different anthocyanins, LA, PA, and RA, were relatively similar, which is attributable to the structural similarities among LA, PA, and RA, particularly in their functional groups [25]. During the film-forming process, the differences in the peak intensities of the bands were relatively small, indicating that no chemical interactions occurred [26]. With an additional amount of 1% being relatively low, some weaker absorption peaks might be masked or difficult to observe.

#### 3.2.3. XRD Patterns

**Figure 5:**
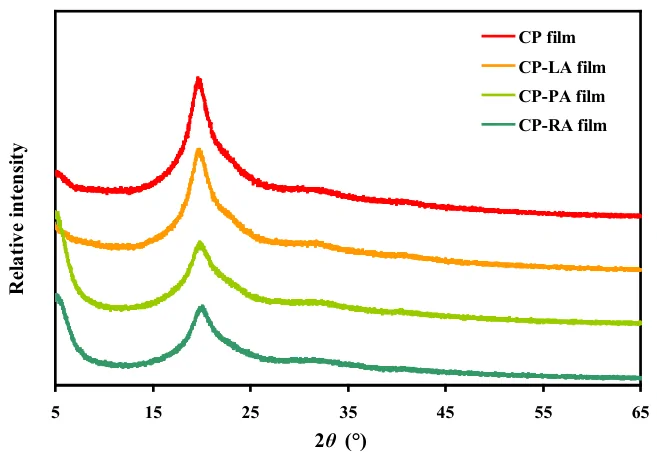
XRD patterns of CP film, CP-PA film, CP-LA film, CP-RA film.

The crystal structures of molecules in films containing different kinds of anthocyanins were examined using XRD (Bruker, Karlsruhe, Germany). Figure 5 shows that the CP film’s diffraction curve has a broad peak at 2*θ* = 19.65°, indicating an ordered structure. The presence of this characteristic peak demonstrated that the arrangement of molecules in the CP film had a certain crystallinity [27]. The diffraction curves of the resulting films following the addition of several types of anthocyanin remained similar to the CP film, suggesting that the anthocyanin addition did not significantly change the crystal structure of the CP/PVA blend matrix, thereby proving the good compatibility between anthocyanins and the CP matrix. In the films with added anthocyanins, the intensity of the diffraction peak showed a gradually decreasing trend. This phenomenon may result from the non-covalent interactions formed between anthocyanin molecules and the CP/PVA matrix; such interactions may interfere with the regular stacking of CP molecular chains, which might be the potential reason for the decreased crystallinity of the system. Such interactions reduced the ordered arrangement among the molecules in the film, thus influencing the intensity of the diffraction peak [28]. According to the experiments mentioned above, the addition of anthocyanins was compatible with CP/PVA and increased the electrostatic contact forces between the molecules in the film matrix. Moreover, the results of FT-IR also confirmed the existence of interactions between anthocyanins and CP/PVA molecules, which further supported the compatibility between anthocyanins and the CP film matrix.

### 3.3. Physicochemical Indexes of the Films

#### 3.3.1. Color and Transmittance

According to Table 1, the CP-LA, CP-PA and CP-RA film matrices exhibited different color characteristics while maintaining relatively high transparency. The CP film without added anthocyanins was colorless, whereas the CP-LA film showed a light pink color, the CP-PA film appeared as a pale yellowish-pink, and the CP-RA film had the most prominent color, presenting a deep pink. The findings indicated that the CP-PA film had the highest values of b* and ΔE*, while having the lowest value of a*, which led to the film showing a yellowish hue. In contrast, the CP-RA film had the highest value of a*, suggesting the deepest color saturation. The order of the total contents of the three kinds of anthocyanins was: RA > LA > PA. Among them, the CP-LA film presented a relatively light pink color due to containing a moderate amount of total anthocyanins. Generally, a higher concentration results in a darker-colored film [29,30,31]. Furthermore, Zhang et al. [32] also observed that although the same amount of roselle anthocyanin was added, the PVA and CP films exhibited distinct coloration.

**Figure 6:**
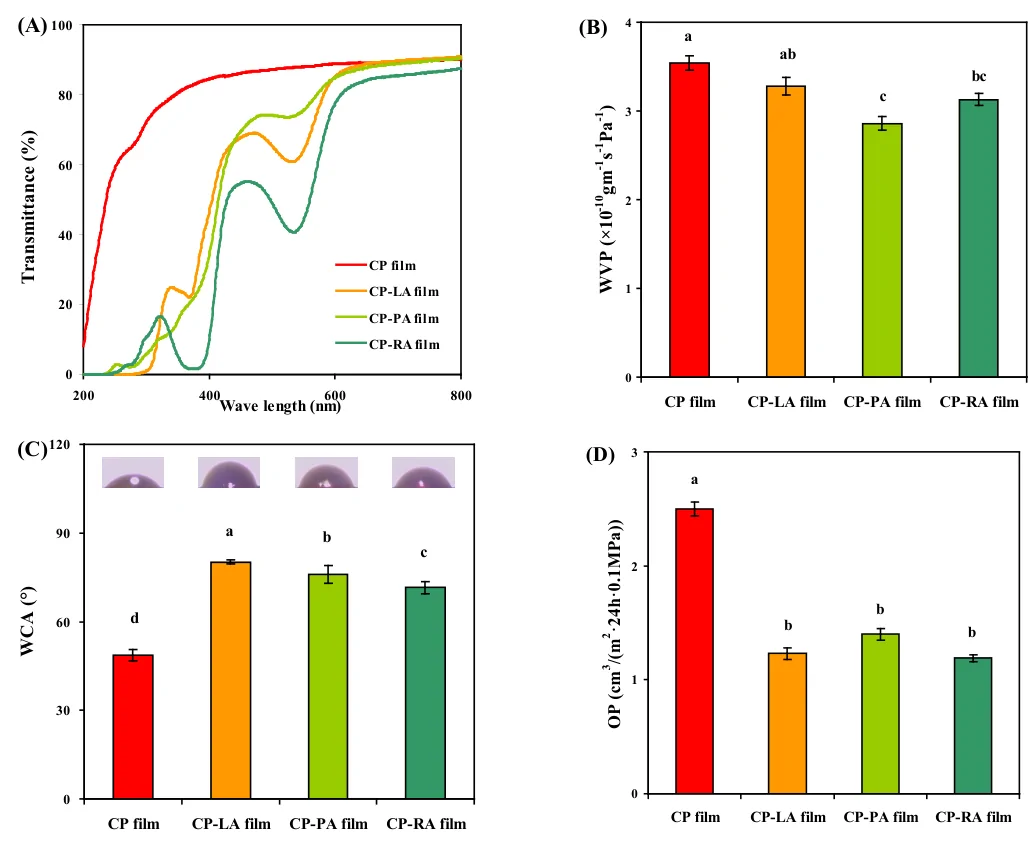
Transmittance (**A**), water vapor permeability (WVP) (**B**), water contact angle (WCA) (**C**) and oxygen permeability (OP) (**D**) of CP film, CP-PA film, CP-LA film, and CP-RA film. Note: Different letters within Figure (**B**–**D**) are significantly different at the *p* < 0.05 level.

Ultraviolet-visible spectroscopy analysis was used to assess the films’ light transmittance performance. The results in Figure 6A showed that the CP film without added anthocyanins exhibited the highest light transmittance. However, adding anthocyanins considerably reduced the transmittance of the CP/PVA matrix films in the ultraviolet-visible light region. This phenomenon was related to the significant absorption ability of the polyphenolic compounds included in LA, PA, and RA towards ultraviolet light, thus increasing the ultraviolet-blocking performance of the CP/PVA matrix films, which enhanced the ultraviolet-blocking efficiency of the CP/PVA matrix films [33]. In the ultraviolet spectrum, there was a negative correlation between the total anthocyanin content of the CP-LA, CP-PA, and CP-RA films and the order of their light transmittance, that is, RA > LA > PA. In particular, the CP-RA film, which contained the highest concentration of total anthocyanins, exhibited the lowest light transmittance at a wavelength of about 560 nm. Furthermore, the film thickness is a critical parameter, as it directly affects the light transmittance [34].

#### 3.3.2. Barrier Properties of the Films

As shown in Table 1, a comparative analysis of the film thicknesses was conducted between the CP-LA, CP-PA, and CP-RA films and the CP film without added anthocyanins. The results demonstrated that although anthocyanins were added, the thickness changes in each film were not significant since the weight ratio of anthocyanins to the CP/PVA matrix was only 1%. This could be attributed to the fact that the low concentration of anthocyanins (1 wt%) had only a modest effect on film thickness. As can be seen from Figure 6B, when anthocyanins were added, the CP film’s WVP decreased substantially. This phenomenon is associated with the internal structural characteristics of the film. We speculate that the anthocyanin molecules might form a relatively stable non-covalent cross-linked network within the system by interacting with the hydrophilic groups in the membrane matrix [35]. At the same time, it can effectively extend the diffusion path of water vapor molecules through the film, increase the permeation resistance, and thus reduce the WVP. The CP-PA film exhibited the lowest WVP value, indicating that there was a more excellent interaction between PA and the CP/PVA matrix, which could more effectively prevent water vapor permeation. It should be noted that in this study, the WVP value was calculated using a formula, where *x* represents the measured thickness of the film. Therefore, the reported WVP value has eliminated the influence of thickness differences and reflects the inherent water vapor barrier performance of the material.

Furthermore, compared with the CP film, the WCA of the CP-LA, CP-PA, and CP-RA films (Figure 6C) was significantly increased. These observations suggest that the formed non-covalent interaction network may occupy some of the hydrophilic groups, thereby potentially reducing the number of sites where the surface interacts with water molecules. This might be the reason for the increase in WCA. This inference still requires more direct surface chemical characterization to be verified. The improvements in both WVP and WCA jointly confirmed that anthocyanins could strengthen the intermolecular interactions within the film matrix and consequently enhance barrier properties.

Furthermore, the CP/PVA matrix may undergo physical binding with the polyphenolic compounds in the anthocyanins, which might help in the more compact arrangement of the chain-like structure and potentially reduce the oxygen permeability (Figure 6D). Meanwhile, these polyphenolic compounds also demonstrated good oxygen-scavenging ability [36]. In particular, the CP-RA film had the lowest OP value among the three, whereas LA, PA, and RA all successfully improved the CP film’s oxygen barrier qualities. This result might be associated with the relatively high total anthocyanin content in the CP-RA film as well as the closer interaction among the film matrix, which is consistent with the finding of Khan et al. [37].

#### 3.3.3. Mechanical Properties of the Films

**Figure 7:**
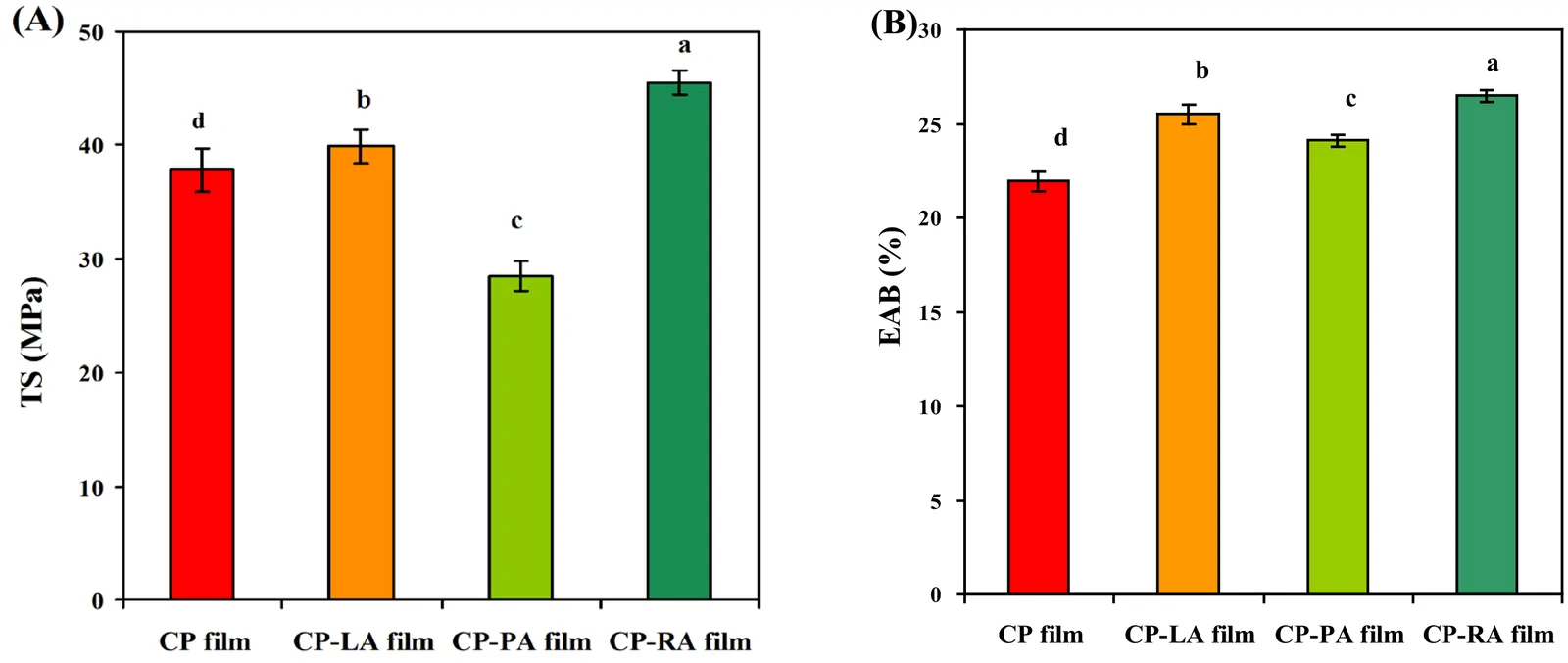

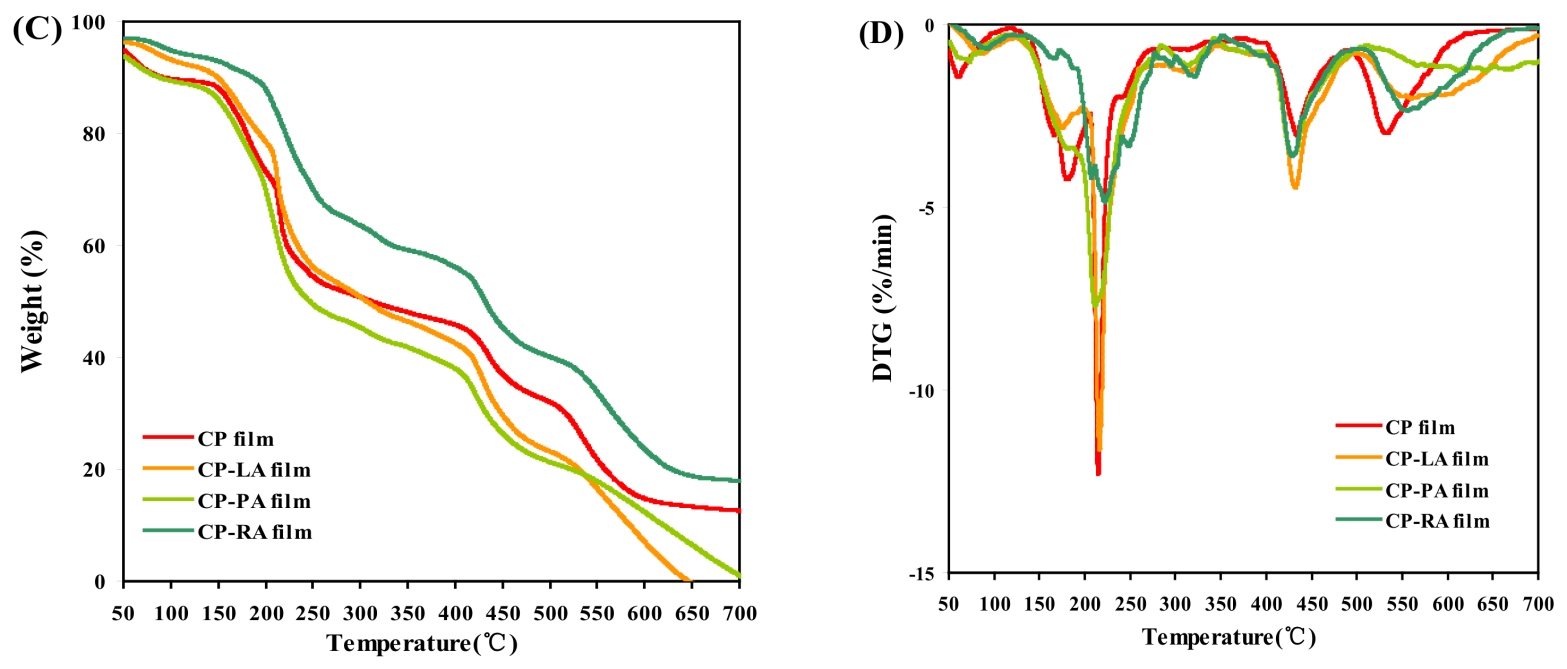
Tensile strength (TS) (**A**), elongation at break (EAB) (**B**), weight loss (**C**) and derivative thermogravimetry (DTG) (**D**) of CP film, CP-PA film, CP-LA film, and CP-RA film. Note: Different letters within figure (**A**,**B**) are significantly different at the *p* < 0.05 level.

The two most important metrics for assessing the mechanical characteristics of films are tensile strength and elongation at break. According to the research by Cinelli et al. [38] and others, starch films’ mechanical strength can be increased by adding PVA, and their flexibility can be enhanced by adding glycerol. In our study, it was found that after the addition of different kinds of anthocyanins, the films had much better elongation at break and tensile strength (Figure 7A,B). From a molecular perspective, this uniformly distributed non-covalent cross-linking network may exert a physical cross-linking effect, linking relatively loose polymer chains into a more compact and uniform whole, and restricting the movement of molecular segments. This might be a potential reason for the increase in the tensile strength of the film [39]. The insertion of RA molecules between the chains partially disrupts the ordered arrangement of the polymer chains, increasing chain mobility, making the chain segments easier to stretch and slide, thus enhancing elongation at break. However, studies by Yong et al. [10] indicate that the mechanical properties of the films are mainly influenced by the nature of the polysaccharides, which differs from the results of this study. This difference may be due to the different polysaccharide matrices used, as the differences in the polysaccharide matrices directly lead to changes in the mechanical properties of the films.

It is important to note that the order of elongation at break and tensile strength was CP-RA film > CP-LA film > CP-PA film. This result indicates that, compared with other extracts, PA significantly improved the films’ tensile strength and elongation at break and had the strongest interaction with the CP/PVA film matrix. However, the research by Yong et al. [10] highlighted that the characteristics of polysaccharides had a major impact on the films’ mechanical properties. This differed somewhat from the research’s findings. This discrepancy might stem from the differences in the polysaccharide matrices used, due to the fact that changes in the polysaccharide matrices will directly affect the films’ mechanical properties. Therefore, the mechanical properties of films are not only affected by the types of anthocyanins but also significantly depend on the structure of the polysaccharide matrices.

#### 3.3.4. Thermal Properties of the Films

Three separate phases were evident in the films’ weight loss process, as illustrated in Figure 7C: the first stage occurred between 50 and 105 °C, the second stage occurred between 106 and 250 °C, and the third stage occurred above 250 °C [40]. The minor mass loss seen below 105 °C was mostly due to the evaporation of physically adsorbed water in the films. Subsequently, in the range from 106 to 250 °C, the further mass loss resulted from the volatilization of glycerol. When the temperature exceeded 250 °C, the anthocyanin extracts and the CP/PVA matrix’s thermal breakdown were the primary causes of the mass loss.

Analysis of the degradation rates of the film samples revealed that at lower temperatures, all films exhibited minimal mass change. As the temperature increased, the films began to decompose. Throughout this process, the degradation rate of the CP-RA film was relatively gentle, indicating that the thermal stability of RA is superior to that of LA and PA. This could be attributed to the formation of strong intermolecular interactions between RA and the CP/PVA (CP) film matrix molecules, which necessitates higher thermal energy to break these interactions. Therefore, the research findings demonstrate that the incorporation of anthocyanin extracts can effectively enhance thermal stability. Ma et al. [41] found that the film structure with the addition of RPPE and ε-PL was denser and exhibited significantly enhanced thermal stability. This result is in agreement with the findings of this experiment.

Moreover, from the maximum peaks of the derivative thermogravimetry (DTG) curves (Figure 7D), the addition of anthocyanin extracts from different plant sources resulted in variations. This indicates that the incorporation of anthocyanin extracts exerts an influence on the weight loss rate of the CP/PVA film matrix, with anthocyanin extracts from different plant sources affecting the thermal properties. Specifically, the CP-RA film’s primary peak emerged at a higher temperature, further confirming its relatively superior thermal stability [42].

### 3.4. Antioxidant Activity of the Films

**Figure 8:**
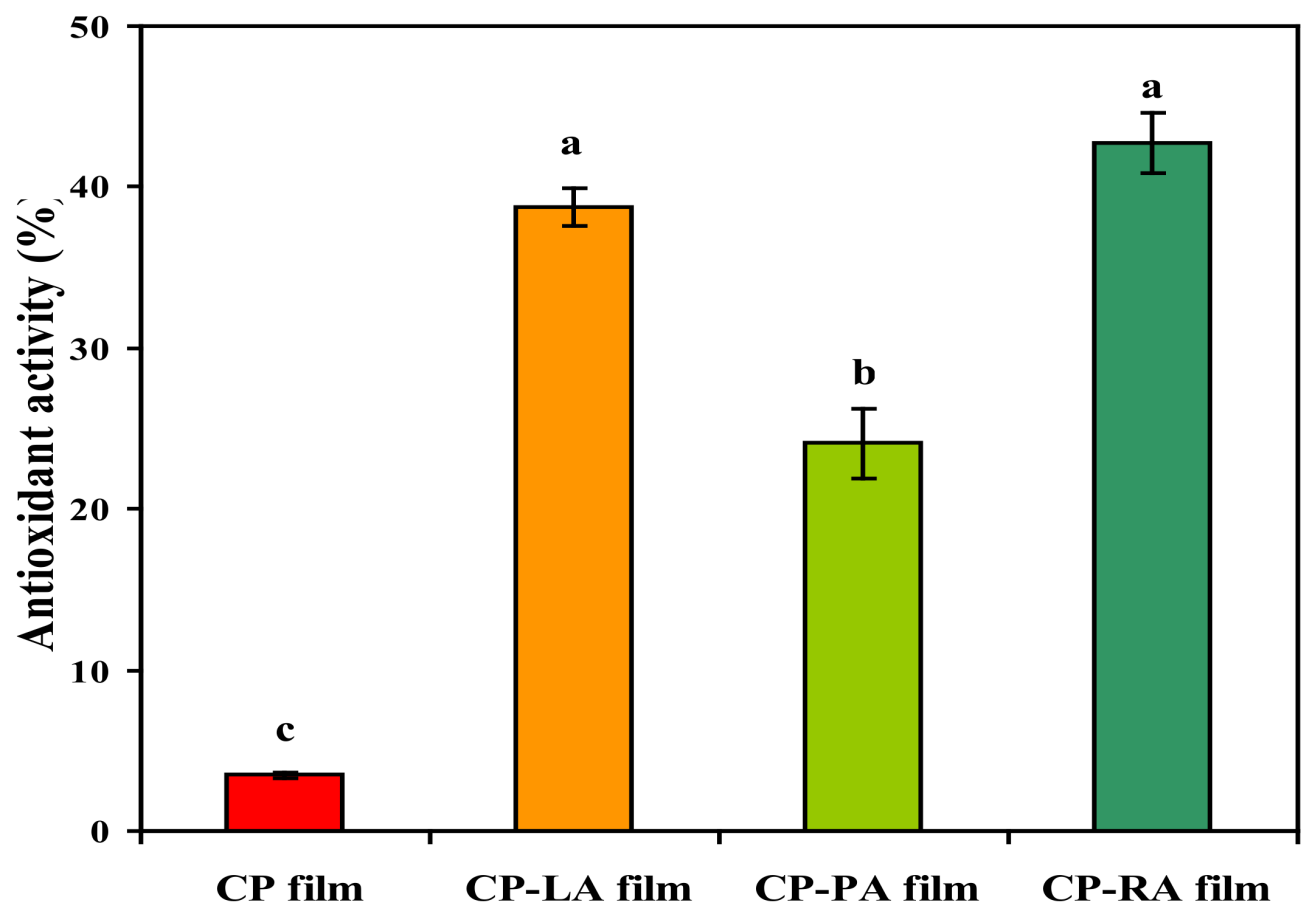
Antioxidant activity of CP film, CP-PA film, CP-LA film, CP-RA film. Note: Values are given as mean ± SD (*n* = 3). Different letters within the figure are significantly different at the *p* < 0.05 level.

The antioxidant performance of the film was evaluated by the DPPH free radical scavenging method in the experiment. The source, quantity, and interactions of anthocyanins with the film matrix all had a substantial impact on the antioxidant activity of the anthocyanin-based films [43]. DPPH free radical is frequently employed as a standard substrate to assess an antioxidant’s antioxidant activity, which is achieved by measuring the scavenging ability of antioxidants towards DPPH free radicals [31]. Anthocyanins contain abundant phenolic hydroxyl groups, which can effectively eliminate free radicals [44]. As shown in Figure 8, the CP film without added anthocyanins exhibited extremely low DPPH free radical scavenging activity. However, after the addition of anthocyanins, this ability was significantly enhanced, and there were differences in the scavenging activities among the blend films with different anthocyanins. The CP-PA film had the lowest scavenging activity, while the CP-RA and CP-LA films showed good scavenging activities. The anthocyanin extracts’ overall phenolic content might directly affect this result. The amount of phenolic compounds in petals is indicated by the total phenolic content, and these compounds have significant antioxidant properties. The total phenolic contents of LA, RA, and PA were 98.90 ± 0.07^b^ mg/g, 102.01 ± 0.15^a^ mg/g, and 68.64 ± 0.22^c^ mg/g, respectively. The contents in the petals of RA and LA were higher than those of PA, meaning that the power to capture free radicals and eliminate them is stronger [45]. The total phenol content is not the only factor determining the clearance rate. The molecular structure of anthocyanins also plays a crucial role. RA is rich in rubiadin-type anthocyanins, whose phenolic hydroxyl structure in the B ring has a low oxidation potential and a fast electron-donating ability, resulting in the highest clearance rate. LA has a total phenol content similar to RA, but both phenolic hydroxyl-type and non-phenolic hydroxyl-type anthocyanins coexist, and its clearance rate is in the middle range; PA has a low total phenol content and only one phenolic hydroxyl group in the B ring, resulting in the slowest clearance rate. The ranking of membrane antioxidant activity is the result of the superposition of the two factors of total phenol content and phenolic hydroxyl configuration.

### 3.5. pH Sensitivity and Ammonia Sensitivity

**Figure 9:**
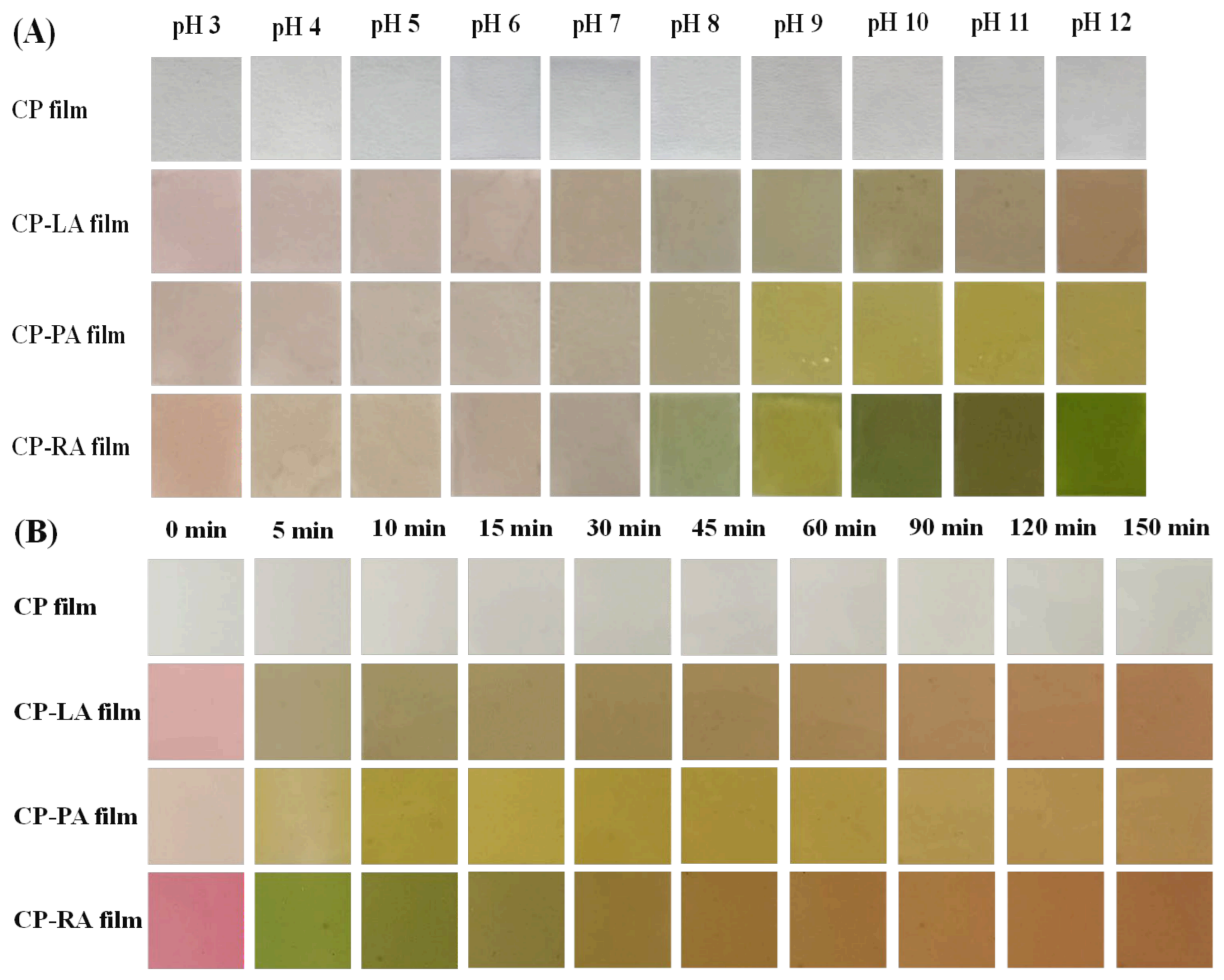
Color changes in CP film, CP-PA film, CP-LA film, and CP-RA film in pH buffer solution (**A**) and in volatile ammonia (**B**).

Various pH levels cause the color to shift. In Figure 9A, after a minute of immersion in buffer solutions with the appropriate pH values, the produced CP/PVA/anthocyanin mix films were removed and blotted dry to document the color changes. While CP-PA film altered from pale pink to yellow, CP-LA film moved from pale pink to light brown, and CP-RA film saw a more noticeable color shift from pink to either brown or yellow-green. The extent of these color shifts was correlated with the structural differences among the anthocyanins from different sources [46]. The substantial color changes observed in different pH buffer solutions are consistent with the findings of Tadele et al. [21], who reported similar pH-responsive behavior in starch-gelatin-anthocyanin intelligent packaging films.

Meat’s freshness can be determined using volatile nitrogen molecules that are created when it spoils. In the investigation modeling the sensitivity to volatile nitrogen compounds, to test the films’ ammonia sensitivity, they were subjected to ammonia gas [47]. The results (Figure 9B) revealed that the only films that clearly altered color when exposed to ammonia gas were CP-LA, CP-PA, and CP-RA.

Specifically, when the light-yellow CP-PA coating turned dark yellow, the colors of the CP-LA and CP-RA films transformed from pink into yellow-green and brown, respectively. This change may be connected to the creation and reorganization of hydroxyl groups in anthocyanin structures [25]. In particular, the CP-RA film demonstrates its potential application value in detecting the degree of spoilage of meat products. Under exactly the same pH, light exposure and imaging conditions, the color transformation of the CP-RA membrane can clearly distinguish the other membranes, and this visually distinguishable trend is completely consistent with the ranking of the total anthocyanin content and total phenolic content of the three membranes.

### 3.6. Color Stability

**Figure 10:**
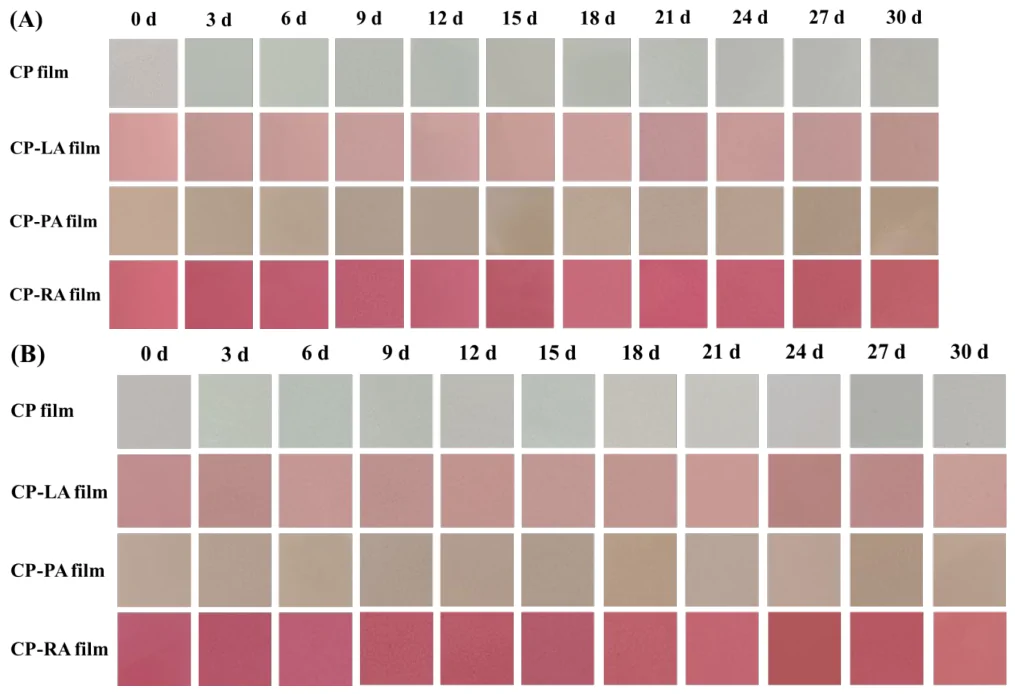
The color change in CP film, CP-PA film, CP-LA film, and CP-RA film under different storage times at 4 °C (**A**) and 20 °C (**B**).

**Figure 11:**
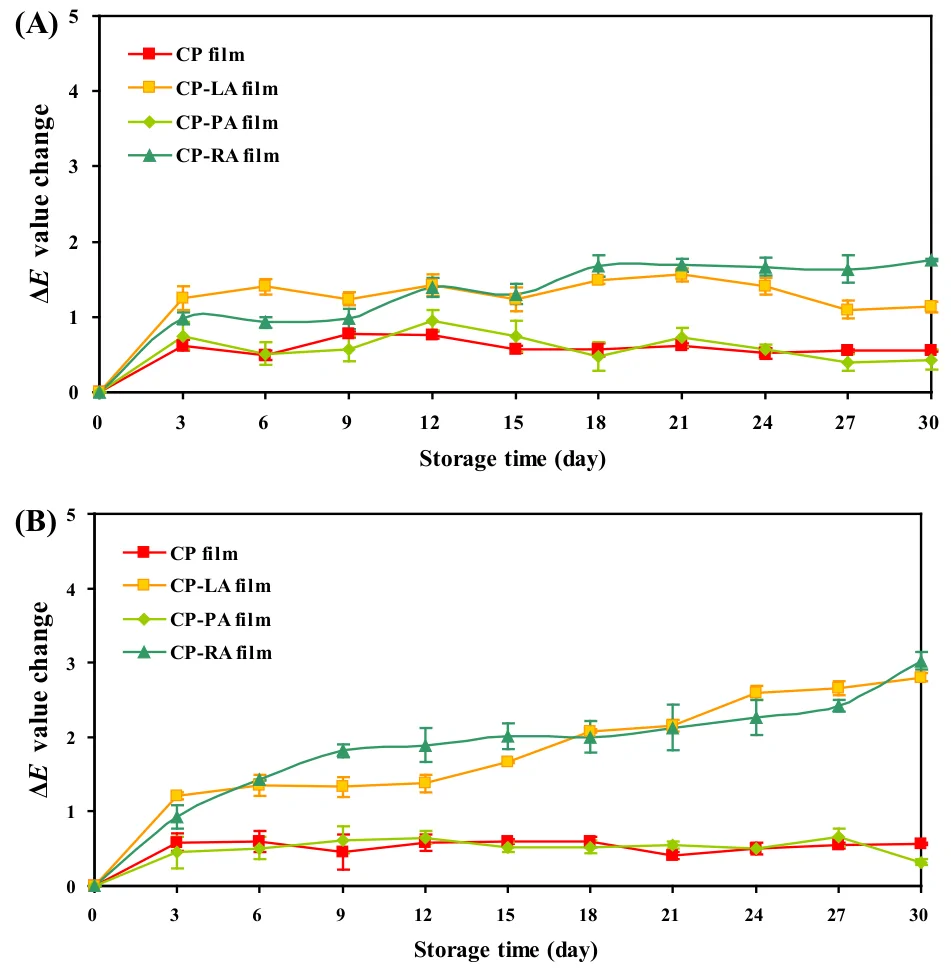
Δ*E* values of CP film, CP-PA film, CP-LA film, and CP-RA film at 4 °C (**A**) and 20 °C (**B**).

This study investigated the color stability of CP/PVA films containing three distinct anthocyanin types over a 30-day storage period at two different temperatures (4 °C and 20 °C) [48]. Figure 10 records the entire process of change, whereas Figure 11 measures the Δ*E* values of the films to assess discoloration.

According to the experimental findings, the CP/PVA films containing different kinds of anthocyanins exhibited significant differences in color stability. When the temperature was 4 °C, the CP film’s color without added anthocyanins remained unchanged, while the CP-RA film’s color shift was relatively noticeable starting from the 15th day, and the Δ*E* value increased accordingly. In contrast, the color fluctuations in the other two films were relatively small, demonstrating relatively good color stability. The color variations in the CP-LA and CP-RA films were more noticeable at 20 °C, gradually showing a transition from yellow to brown, and as time went on, the Δ*E* values progressively increased. On the contrary, at both 4 °C and 20 °C, the CP-PA film’s color barely altered while being stored.

These findings confirmed that elevated temperature and light levels may hasten anthocyanin breakdown, thereby impacting the films’ color stability [48]. The reasons for the color changes might be anthocyanin degradation [49]. Comprehensive analysis showed significant advantages in terms of the color stability of anthocyanins. Its color changes were distinct and easy to recognize. This characteristic endows the CP-RA film with a relatively high application potential in monitoring environmental changes, particularly in circumstances such as variations in pH and the volatile ammonia present.

Based on the comprehensive results of SEM, FTIR, XRD and various physical and chemical functional tests, it can be observed that the three flower-based composite films have significant differences in their microstructure, barrier properties, mechanical strength, and pH color sensitivity. However, it is necessary to objectively state that since the entire study compared anthocyanins from different flower sources and lacked component characteristic analysis, it was difficult to determine whether the observed differences were due to the composition of anthocyanins, the co-extracted phenolic substances, or other components. Future research will prepare sufficient samples and use LC-MS technology to analyze the component differences, further clarifying the contribution of each component to the film performance.

### 3.7. Freshness Monitoring of Pork Ball Using CP-PVA-Anthocyanin Film

During the spoilage process of animal-derived foods, the emission of volatile nitrogen-containing chemicals is common. This raises the pH level inside the container. Customers can make an intuitive assessment of the freshness of Pork balls by using the indicator films’ color variations [50,51]. Different kinds of anthocyanin/CP/PVA films were used in the experiment on the spoilage degree of Pork balls to compare the monitoring capabilities of different anthocyanin films for the freshness of Pork balls.

**Figure 12:**
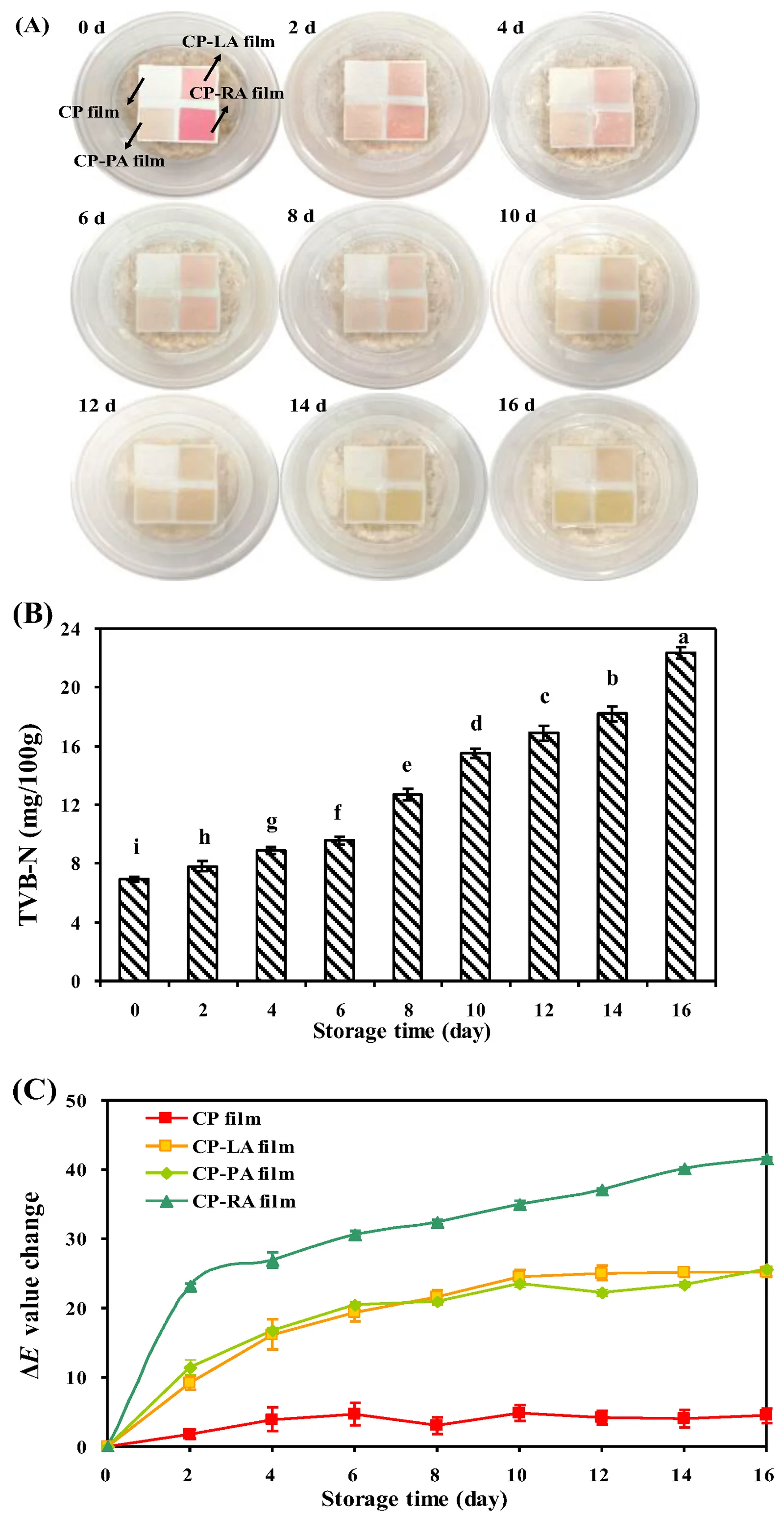
Color changes (**A**), TVB-N values (**B**) and Δ*E* values (**C**) of CP film, CP-PA film, CP-LA film, and CP-RA film. Different letters within the (**B**) are significantly different at the *p *< 0.05 level.

As illustrated in Figure 12, with the extension of time, all the anthocyanin films displayed obvious color shifts: gradually changing from the initial pink to the final yellow or yellowish-green. The CP-RA film’s color changed most dramatically, going from deep pink to yellow-green. As the amount of time spent in storage grew, the TVB-N value (Figure 12B) in the Pork balls also gradually increased. Ten days later, the value reached 15.49 mg/100 g, surpassing Chinese Standard GB 2707-2016 [52]’s safety limit (15 mg/100 g), indicating that the Pork balls were no longer fresh. Alterations in the films’ Δ*E* values similarly reflected changes in their color. As time went by, these three distinct types of anthocyanin films’ Δ*E* values increased significantly. Among them, during the spoilage process of Pork balls, the CP-RA film had the largest change in the Δ*E* value, indicating that it was the best choice for monitoring the Pork balls’ freshness. As shown in Table 2, the storage time and the type of film have a highly significant impact on the Δ*E* values (*p* < 0.01), indicating that both of them are key factors influencing the Δ*E* values.

**Figure 13:**
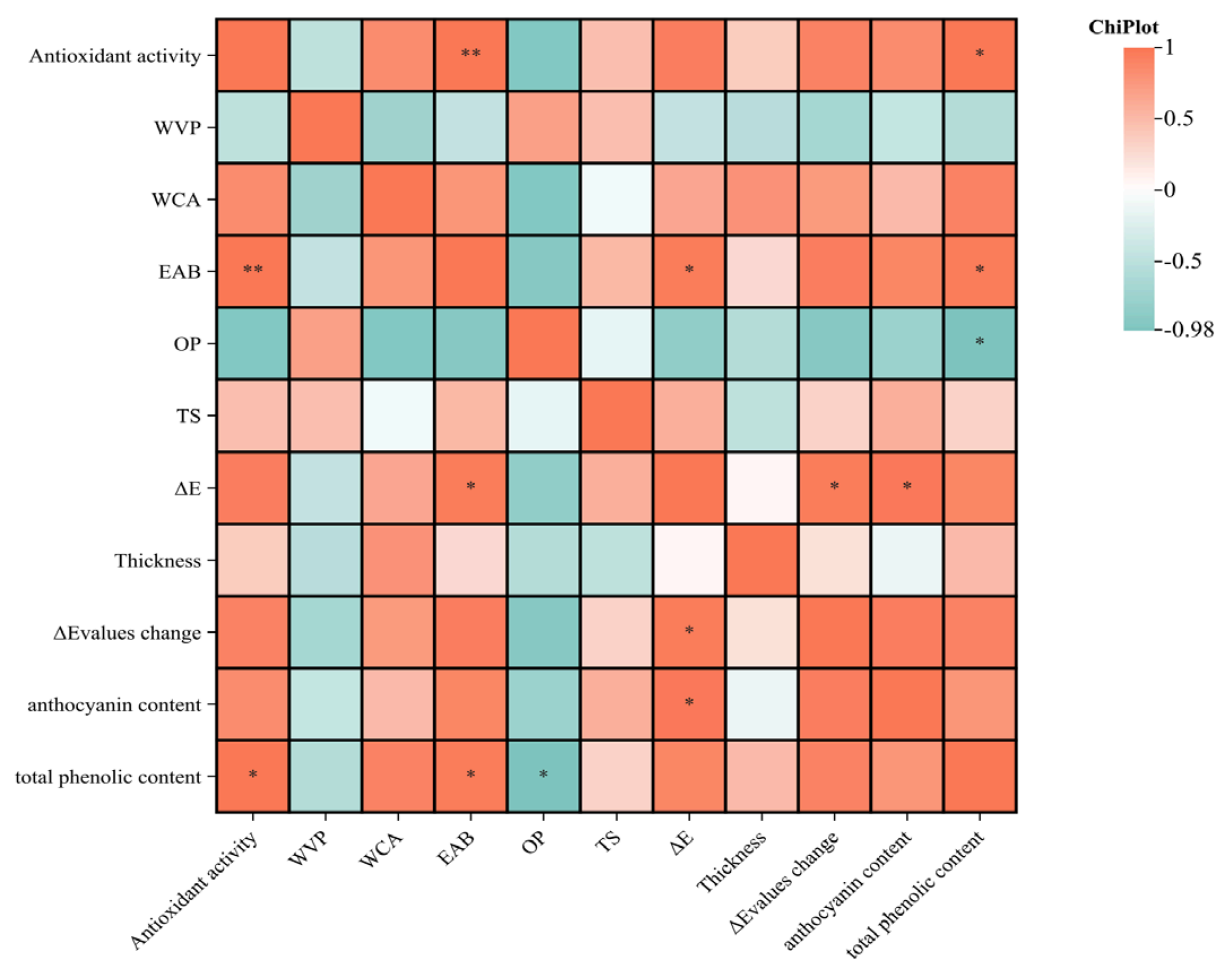
A correlation analysis among anthocyanin content, total phenolic content, antioxidant activity, barrier properties, mechanical properties, and freshness monitoring performance. Note: *, *p* < 0.05; **, *p* < 0.01.

Figure 13 was analyzed via Pearson correlation analysis. Significant positive correlations were identified among anthocyanin content, total phenolic content and antioxidant activity. It was demonstrated that phenolic compounds were the core contributors to the antioxidant capacity of the system, and the free radical scavenging activity of the films was directly determined by anthocyanins as characteristic polyphenols. A negative correlation was observed between WCA and WVP. The penetration of water molecules could be inhibited when the surface hydrophobicity of films was improved, and the water vapor barrier property was thereby enhanced. Significant positive correlations were detected between total phenolic content and both Δ*E* and Δ*E* value changes. It was illustrated that the pH colorimetric response sensitivity of films could be enhanced by high polyphenol loading. Meanwhile, the oxidative degradation of anthocyanins was retarded by the antioxidant protective effect of polyphenols, and the stability of the indicator function was therefore improved.

## 4. Conclusions

This study aims to extract anthocyanins from the petals of three ornamental flowers (LA, PA, RA) and prepare composite membranes with CP/PVA. The main purpose is to evaluate the structural, physicochemical and functional properties of these membranes and to achieve visual monitoring of the freshness of Pork balls. Additionally, different types of anthocyanins affect the barrier properties, mechanical properties, thermal properties, antioxidant capacity and pH reaction ability of the indicator films. Through systematic screening of the three flower species investigated in this study, RA was identified as a promising source, with relatively high total anthocyanin and total phenolic contents. The CP-RA film consequently exhibited favorable antioxidant activity, light/oxygen barrier properties, and pH-responsive color-changing performance compared with the other two formulations. Furthermore, few studies have adopted this flower source film to monitor the freshness of Chinese-style pork balls. This study, conducted under laboratory conditions, preliminarily verified the potential feasibility of using this flower-derived indicator for real-time evaluation of cooked food products. In conclusion, this study provides a novel idea for the preparation of intelligent indicator films from natural flower source materials, which can offer certain references for the subsequent development of packaging for Chinese-style meat products.

The monitoring experiment of Pork balls in this study mainly relied on parameters such as total volatile basic nitrogen (TVB-N) and pH, but lacked direct verification of microorganisms. Systematic verification will be conducted in the future. This study was limited by the initial sample size, resulting in some experiments being unable to be replicated and verified. Moreover, no precise color parameters under each pH condition were provided in the methods. In the future, larger batches of samples will be prepared and reserved as backups. At the same time, color parameter analysis will be supplemented to ensure the integrity and reproducibility of the research. Future studies should include HPLC and LC-MS analyses to identify and quantify the anthocyanin components, which were not covered in this work.

## Figures and Tables

**Table 1:** Appearance, color and thickness of CP film, CP-PA film, CP-LA film, CP-RA film.

Films	Appearance	L*	a*	b*	ΔE*	Thickness (mm)
CP film	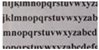	91.25 ± 0.42 ^a^	0.52 ± 0.73 ^d^	−1.82 ± 0.4 ^d^	4.47 ± 0.37 ^d^	0.105 ± 0.009 ^b^
CP-LA film	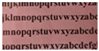	75.61 ± 0.24 ^c^	22.09 ± 0.81 ^b^	4.97 ± 0.86 ^c^	26.57 ± 0.15 ^b^	0.114 ± 0.009 ^a^
CP-PA film	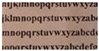	81.91 ± 0.18 ^b^	6.94 ± 0.11 ^c^	11.10 ± 0.29 ^a^	19.10 ± 0.26 ^c^	0.113 ± 0.007 ^a^
CP-RA film	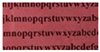	64.32 ± 0.29 ^d^	43.07 ± 0.24 ^a^	7.01 ± 0.34 ^b^	43.43 ± 0.35 ^a^	0.106 ± 0.005 ^b^

Note: Values are given as mean ± SD (*n* = 3). Data with different letters in the same column mean significant difference (*p* < 0.05).

**Table 2:** Two-way ANOVA by univariate during storage for two storage days.

Analysis of Variance Table					
Source of Variation	The Sum of the Squares of III	df	MS	*F*	Significance
storage time	1859.948	8	232.494	10.32	<0.001
variety	3060.191	3	1020.064	45.281	<0.001
error	540.659	24	22.527		
total	5460.798	35			

## Data Availability

The original contributions presented in this study are included in the article. Further inquiries can be directed to the corresponding author.
